# Stabilizing an ultrathin MoS_2_ layer during electrocatalytic hydrogen evolution with a crystalline SnO_2_ underlayer†

**DOI:** 10.1039/d1ra00877c

**Published:** 2021-05-18

**Authors:** Jonas Englhard, Yuanyuan Cao, Sebastian Bochmann, Maïssa K. S. Barr, Stéphane Cadot, Elsje Alessandra Quadrelli, Julien Bachmann

**Affiliations:** Chemistry of Thin Film Materials, Department of Chemistry and Pharmacy, IZNF, Friedrich-Alexander University of Erlangen-Nürnberg Cauerstr. 3 91058 Erlangen Germany julien.bachmann@fau.de; C2P2 UMR 5265, Université de Lyon, Institut de Chimie de Lyon, CNRS, Université Lyon 1, ESCPE Lyon 43 Bd. du 11 Novembre 1918 69616 Villeurbanne France; Institute of Chemistry, Saint Petersburg State University Universitetskii pr. 26 198504 St. Petersburg Russia

## Abstract

Amorphous MoS_2_ has been investigated abundantly as a catalyst for hydrogen evolution. Not only its performance but also its chemical stability in acidic conditions have been reported widely. However, its adhesion has not been studied systematically in the electrochemical context. The use of MoS_2_ as a lubricant is not auspicious for this purpose. In this work, we start with a macroporous anodic alumina template as a model support, add an underlayer of SnO_2_ to provide electrical conduction and adhesion, then provide the catalytically active, amorphous MoS_2_ material by atomic layer deposition (ALD). The composition, morphology, and crystalline or amorphous character of all layers are confirmed by spectroscopic ellipsometry, X-ray photoelectron spectroscopy, grazing incidence X-ray diffractometry, scanning electron microscopy and energy dispersive X-ray spectroscopy. The electrocatalytic water reduction performance of the macroporous AAO/SnO_2_/MoS_2_ electrodes, quantified by voltammetry, steady-state chronoamperometry and electrochemical impedance spectroscopy, is improved by annealing the SnO_2_ layer prior to MoS_2_ deposition. Varying the geometric parameters of the electrode composite yields an optimized performance of 10 mA cm^−2^ at 0.22 V overpotential, with a catalyst loading of 0.16 mg cm^−2^. The electrode's stability is contingent on SnO_2_ crystallinity. Amorphous SnO_2_ allows for a gradual dewetting of the originally continuous MoS_2_ layer over wide areas. In stark contrast to this, crystalline SnO_2_ maintains the continuity of MoS_2_ until at least 0.3 V overpotential.

## Abstract 2

A molybdenum disulfide coating deposited on a macroporous substrate as an electrocatalyst is mobile on an underlying amorphous tin dioxide substrate, but remains continuous and impervious to acidic conditions on crystalline tin dioxide.

## Graphical abstract

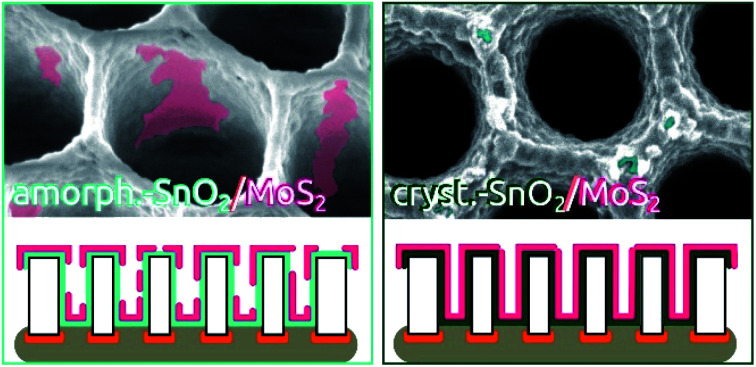

## Introduction

With increasing contributions of renewable, inherently intermittent, energy sources to the energy mix, finding a way to store excess electrical power becomes crucial to the success of the energy transition.^1,2^ Electrolysis represents a prominent solution, which stores electrical power in chemical form, as dihydrogen. This clean fuel affords high energy storage density and can be transported and converted back to electrical energy in fuel cells.^3,4^ To maximize the energy efficiency of the whole storage and release cycle, the electrolysis of water must be performed at low overpotentials, and therefore, be effectively catalyzed on both electrodes. For the hydrogen evolution reaction (HER), noble metals such as Pt or its alloys still offer the lowest overpotentials.^5–7^ Drawbacks are, however, low noble-element abundance and high costs. Thus, the search for alternative catalyst materials is of significant interest.^8^

MoS_2_ is considered as a highly interesting potential alternative to noble metals as an electrocatalyst for H_2_ evolution. It has been widely reported as being chemically stable in acidic conditions, but its (equally important) adhesion to electrode substrates has not been studied systematically.^9–12^ Catalysis at the surface of this layered transition metal dichalcogenide^13^ occurs on S vacancies at the edges of individual sheets, which feature a H adsorption free energy Δ*G*_ads_ comparable to noble metals such as Pt.^14–16^ The basal planes of the crystal are not involved in catalysis, so that amorphous forms of MoS_2_ exhibiting a high density of Mo defects and disulfide sites offer highest HER activity.^17–19^

The electrical properties of amorphous MoS_2_ (a-MoS_2_), however, are mediocre and hinder its scalable application in hydrogen production. This impediment becomes problematic when non-planar electrode substrates are used. Such electrodes offering high specific surface area usually serve to increase macroscopically defined current densities in electrochemical energy conversion devices and feature porous surface. In that perspective, MoS_2_ has already been deposited on carbon nanospheres,^20,21^ CdS nanorods,^22,23^ porous metallic MoO_2_,^24^ and titanium oxide nanotube arrays.^9,25^ In those examples, the current must be transported by an electrically conductive substrate since long distances along thin a-MoS_2_ layers would cause too high ohmic resistance losses.

The study of transport and surface limitations at nanoporous electrode surfaces, and the optimization of geometry towards electrocatalytic turnover, can be performed at a geometrically perfect model system presented by ‘anodized’ aluminum oxide (as the substrate) coated (with the functional layer) by atomic layer deposition. Anodized aluminum oxide (‘anodic alumina’ or AAO) made by electrochemical oxidation of the metal delivers ordered arrays of straight, parallel and cylindrical pores the diameter and length of which can be adjusted accurately and varied systematically (on the scale of 10–500 nm and of 0.5–100 μm, respectively).^26^ To coat substrates with pores of such high aspect ratios, atomic layer deposition (ALD) has emerged as a key technique. Its ability to coat nanoporous substrates with a-MoS_2_ in a homogeneous, conformal manner has been demonstrated.^9,27^ If the electrically insulating AAO is used as a substrate, however, the limited electrical conductivity of a-MoS_2_ coatings of ≤20 nm thickness must be compensated for by an appropriate underlying layer.^28,29^ We propose SnO_2_ in this function, since it has proven to be advantageous for this purpose.^30,31^ Its deposition *via* ALD is well established^32^ and it features a high charge carrier density and overall conductivity.^33^ Additionally, its conduction band is located at about −4.5 eV (*vs. E*_vac_) and matches the respective band of MoS_2_ quite well.^34^ The goal of this study is to establish the conditions in which a macroporous AAO/SnO_2_/a-MoS_2_ electrocatalyst model system is stable. Limitations to stability are defined by MoS_2_ itself and SnO_2_, the Pourbaix diagram of which indicates cathodic corrosion in acidic media.^35^ This corrosion can be prevented by the a-MoS_2_ layer, if it is continuous and remains immobile and pinhole-free during electrocatalytic turnover. We find that the most important aspect is the crystallinity of the SnO_2_ coating, which can fix a-MoS_2_ and prevent degradation.

## Experimental

### Chemicals

Chemicals are of analytical reagent grade, were purchased from Sigma-Aldrich, Alfa Aesar, ABCR, Carl Roth, Fisher Chemicals or VWR and then used as received. The aluminum foils (99.99%) were purchased from Smart Membranes and for anodization purposes, water purified in a Millipore Direct-Q 3 system was used. Silicon (100) wafers with an approximately 200 nm thick oxide layer were acquired from Silicon Materials Inc. H_2_S (3% in N_2_) was purchased from Air Liquide (Germany), the molybdenum precursor Mo[N(Me)_2_]_4_ was synthesized following a literature procedure.^36^ Sn[N(Me)_2_]_4_ was purchased from ABCR (Germany) in 99.99% purity. H_2_O_2_ (30%) was purchased from Carl Roth. In our hands, the consumption of metal–organic ALD precursors is on the order of 1 g per 1000 cycles.

### Preparation of nanostructured composite electrodes

**Fig. 1:**
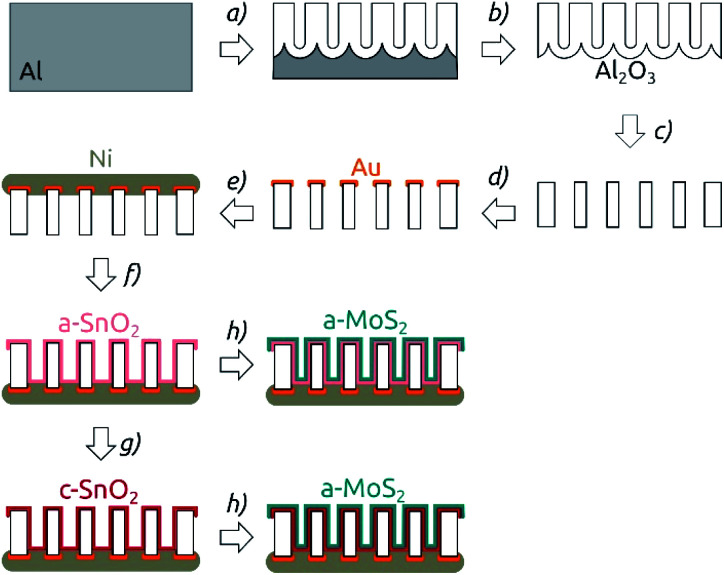
Preparation procedure for nanostructured MoS_2_ electrodes. The scheme shows the cross-section of the membrane. Note that at all steps of the scheme the membrane is still a continuous network, only traversed by pores. (a) Two-step anodization of Al foil. (b) Removal of the remaining Al substrate. (c) Barrier layer removal and pore widening in phosphoric acid. (d) Sputter coating of the Au contact. (e) Electroplating of a Ni backside contact. (f) ALD of SnO_2_. (g) Annealing (4 hours, 400 °C, N_2_). (h) ALD of MoS_2_.

The nanostructured electrodes were prepared in multiple steps as depicted in Fig. 1. Macroporous aluminum oxide membranes were obtained by a standard two-step anodization of aluminum (step (a) in Fig. 1).^37^ The anodization was carried out using home-made four-opening PVC beakers, which held four pieces of Al foil at a time and which were screwed on thick copper plates (serving as the electrical contact). The beakers were filled with electrolyte and equipped with a stirrer including a counter-electrode consisting of Ag wires. Firstly, the aluminum plates were electropolished in a cooled solution of perchloric acid in ethanol (1 : 3 v/v HClO_4_/EtOH) for 5 min under 20 V. Then the solution was rinsed away and the beakers were filled with a solution of 1 wt% H_3_PO_4_ in Millipore water. After cooling to 0 °C using a Unichiller 012-MPC cooler, the Al plates were anodized under a constant voltage of 195 V for 23 h. This somewhat long first anodization duration is chosen so as to optimize the degree of order reached for the second anodization. The disordered porous Al_2_O_3_ obtained was dissolved in a chromic acid solution (0.18 M CrO_3_ in 6 wt% H_3_PO_4_) for 24 h at 45 °C. Then, a second anodization in 1 wt% H_3_PO_4_ at 0 °C was performed for 3, 4, 6, or 8 h, yielding porous anodic alumina with various pore lengths (Fig. 1). Then, a solution of 0.7 M CuCl_2_ in 10% HCl was used to remove the remaining Al underneath the pores (step (b) in Fig. 1). The oxide barrier layer on the extremity of the pores was removed (and the pores were simultaneously widened) by keeping the membranes in 10 wt% H_3_PO_4_ for 37 min (step (c)). A 100 nm thick Au contact was DC-sputter coated on one side of the AAO membranes using a reactor from Torr International Inc. (step (d)). To obtain a thicker contact, this gold layer was then utilized as a cathode to galvanically plate a thick Ni layer. For this purpose, a diluted Watts electrolyte (0.57 M NiSO_4_, 95 mM NiCl_2_, 0.5 M H_3_BO_3_) was used and a potential of −2.3 V was applied for 5 h (Fig. 1e).

The deposition of SnO_2_ was performed by ALD in a commercial Gemstar-6 XT ALD reactor equipped with a Cobra BA 0100 C pump from Busch and with N_2_ as carrier gas (Fig. 1f). Sn[N(Me)_2_]_4_ and H_2_O_2_ were used as precursors and maintained in stainless steel bottles at 65 °C and at room temperature, respectively. For the deposition, the chamber of the reactor was heated up to 150 °C. The tin precursor was pulsed for 0.6 s into the chamber, stayed in there for 50 s (exposure) and was subsequently pumped away by applying a continuous N_2_ flow and vacuum to the chamber for 90 s (purge). In the second half-cycle, H_2_O_2_ was pulsed into the reactor with pulse, exposure and purge times of 0.6 s, 50 s and 70 s, respectively. 64 ALD cycles (c) yielded a deposition of 10 nm SnO_2_, which corresponds to a growth per cycle (GPC) of approximately 1.6 Å per cycle.

After SnO_2_ was deposited, the membranes were optionally annealed in a furnace from Nabertherm under N_2_-atmosphere (Fig. 1g). The temperature was ramped up over 5 h to the target temperature of 400 °C, which was kept for 4 h. The samples were then cooled down to room temperature over 8 h.

On top of the annealed tin oxide layer, MoS_2_ was deposited by atomic layer deposition (ALD) in a home-built hot-wall reactor equipped with a Cobra BA 0100 C pump from Busch and with N_2_ as the carrier gas (step (h) in Fig. 1). Mo[N(Me)_2_]_4_ and H_2_S were used as molybdenum and sulfur sources and kept at 65 °C and at room temperature, respectively.^27^ The deposition was carried out at 95 °C chamber temperature. For Mo[N(Me)_2_]_4_, two pulses with a duration of 0.7 s (3 s apart), an exposure time of total 50 s and a purge time 60 s were used. The H_2_S half-cycle was carried out with pulse, exposure and purge times of 0.2 s, 50 s and 60 s, respectively. 40 c yielded a deposition of 10 nm, corresponding to a GPC of approximately 2.5 Å per c. Thereafter, the samples were stored under N_2_ atmosphere until they were characterized electrochemically.

### Characterization

The layer thicknesses of semiconductors deposited by ALD were determined by spectroscopic ellipsometry (Sentech SENpro equipped with a tungsten halogen lamp) on Si (100) wafers, using the software SpectraRay 3. The deposited semiconductor crystal structures were characterized by measuring grazing incidence X-ray diffraction (GIXRD; incident angle: 0.6°) using a Bruker D8 Advance diffractometer equipped with a Cu K_α_ radiation source and a LynxEye XE-T detector. Scanning electron microscopy (SEM) and energy-dispersive X-ray spectroscopy (EDX) were performed using either a JEOL JSM 6400 PC system implemented with a LaB_6_ cathode and SDD X-ray detector or a Carl Zeiss Gemini 500 field-emission instrument. X-ray photoelectron spectroscopy (XPS) was measured with a monochromatized Al K_α_ source on a PHI Quantera II system. The core level spectra obtained were evaluated using a Shirley background subtraction and Voigt functions (convolution of Lorentz and Gauss distributions) to fit individual peaks. The spectra were all calibrated to a C 1s binding energy position of 284.8 eV.

### Electrochemical studies

After being coated with MoS_2_, the samples were cut into small pieces and glued onto copper plates with conductive double-sided copper tape. Then, the sample pieces were covered with a mask made out of polyimide tape (Kapton®) featuring a laser-cut circular hole with a diameter of 2.0 mm. For the electrochemical measurements, a three-electrode setup featuring a Pt mesh counter electrode and a Ag/AgCl reference electrode (3 M NaCl, standard potential shifted −0.20 V compared to the standard hydrogen electrode SHE) was used. Most of the measurements were carried out in a 0.1 M H_2_SO_4_ solution, whereas 0.5 M H_2_SO_4_ was used in a small of cases. All electrochemical measurements including cyclic voltammetry (CV), linear sweep voltammetry (LSV), electrochemical impedance spectroscopy (EIS) and steady state chronoamperometry were carried out at room temperature on Gamry Interface 1000 potentiostats. Cyclic voltammograms were measured at a scan rate of 50 mV s^−1^. The onset potential for HER was hereby defined as the overpotential required to reach the threshold current density of 10 mA cm^−2^. Chronoamperometric measurements were performed at different applied potentials for 1 h; the resulting average current density was determined by averaging over the last 30 min. Impedance spectroscopy was measured at a potential of −0.3 V (*vs.* SHE) between 100 kHz and 0.02 Hz and the obtained data were fitted using the software Gamry Echem Analyst. The circuit model used accounts for a series resistance (*R*_u_) and an RC element characterizing the liquid/solid interface. This element is modelled *via* a charge transfer resistance *R*_ct_ connected in parallel with a constant-phase element *Q*_ls_, characterized by the impedance *Y* and the capacitor ideality factor *α* (*α* = 1 for an ideal capacitor) and which accounts for the electrochemical double layer.

## Results and discussion

### Preparation and characterization of AAO/SnO_2_/MoS_2_ composite electrodes

**Fig. 2:**
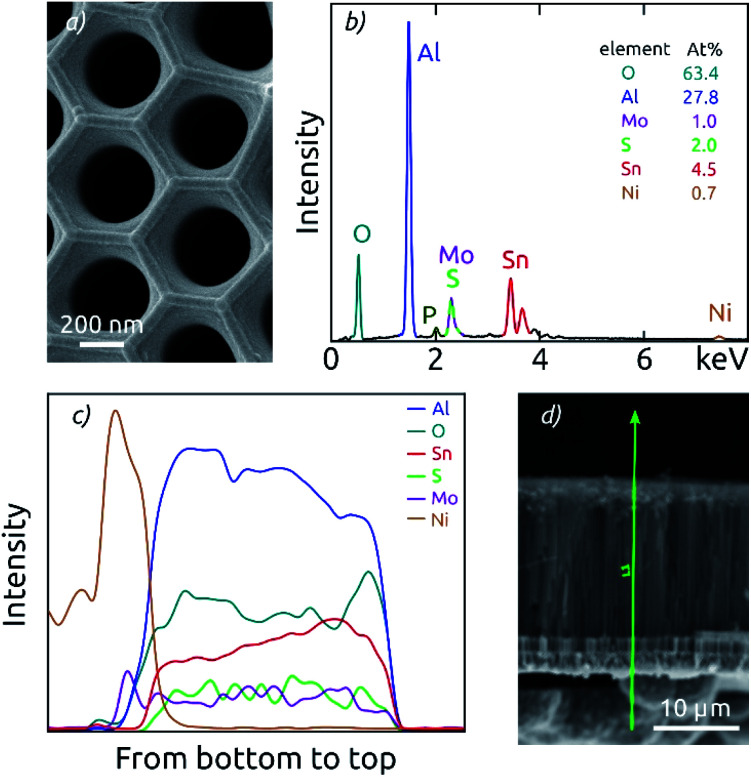
Characterization of an AAO/SnO_2_/MoS_2_ composite electrode with the highest pore length (18.5 μm). (a) Top-view SEM of the macroporous composite electrode. (b) EDX spectrum measured on the top of the sample. (c) Cross-section EDX analysis measured along the pores demonstrates the good uniformity of SnO_2_ and MoS_2_ coatings. The signals of the elements analyzed are arbitrarily scaled. (d) Scanning electron micrograph of the membrane in cross-section view, indicating the EDX measurement path as a green line.

The general procedure for nanostructured MoS_2_ electrode preparation is summarized in Fig. 1. Macroporous AAO substrates are prepared by a two-step anodization of aluminum in phosphoric acid. The second anodization carried out for durations of 3 to 8 h results in pore lengths of 8.6 to 18.5 μm as determined by cross-section scanning electron microscopy (SEM) (Fig. S1†). A pore diameter of ∼380 nm is achieved *via* subsequent pore widening through wet-chemical etching in phosphoric acid (Fig. 2a). One face of the porous samples is then conferred with a metal contact by sputter coating Au (100 nm), followed by electrodeposition of Ni using a Watts electrolyte. This results in a metallic contact of approximately 3 μm thickness (Fig. S1†), which defines the backside of the electrode.

The nanostructured templates are then coated with SnO_2_ by ALD and optionally annealed at 400 °C for 4 h. This temperature is the minimal value needed to yield crystallization at a reasonable rate, and it is the maximum that our samples can tolerate without risking delamination of the Ni contact. In the final step, the substrates are coated with MoS_2_*via* ALD. Spectroscopic ellipsometry and AFM step edge measurements performed on planar reference substrates confirm that a film thickness of around 10 nm is achieved with 40 cycles (c) of MoS_2_ ALD (Fig. S2†), as expected based on the published growth rate.^9^ Importantly, SEM investigation proves that the thin MoS_2_ film on the AAO/SnO_2_ substrate is perfectly continuous (Fig. 2a).

**Fig. 3:**
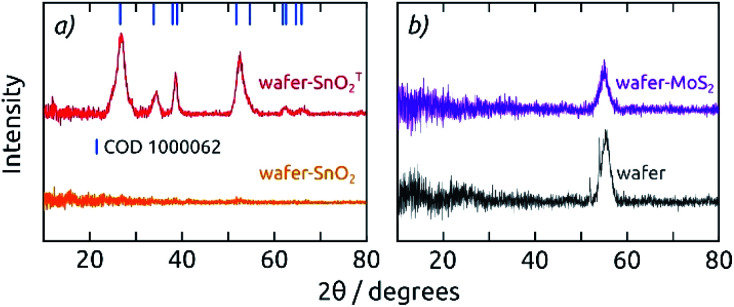
(a) Grazing-incidence X-ray diffraction patterns of 20 nm thick SnO_2_ layers on a Si (100) wafer, as deposited (orange) and annealed (T, red): annealing converts a-SnO_2_ to c-SnO_2_. (b) Grazing-incidence X-ray diffraction patterns of 20 nm as-deposited MoS_2_ on a Si (100) wafer (purple) compared to the bare wafer without coating (black).

EDX analysis performed on the top face of the electrodes confirms the chemical composition of the full stack (Fig. 2b). The AAO template is manifested by the signals of O, Al and P (where P is due to phosphate incorporation into the alumina upon anodization).^26,38^ The thin films deposited generate additional Mo and S signals in an atomic ratio of 1 : 2, as well as Sn peaks.

Cross-section EDX analysis demonstrates a good uniformity of the ALD coatings along the AAO pores of all lengths (Fig. 2c). EDX signals of Al and O are attributed to the AAO template and thus define the full sample thickness. In this section, fairly constant signals are also observed for the elements Mo, S and Sn, which are part of the ALD-grown films. Due to the spectral overlap of the Mo L-line and the S K-line, the attribution of the signal intensity to each element exhibits some uncertainty. However, the sum of both signals is observed to be constant along the pore length. The presence of nickel contact is evident on one side of the sample. Altogether, the EDX results prove that both SnO_2_ and MoS_2_ form a continuous layer from the top to the bottom of the AAO pores, and successfully contact the nickel electrode.

Grazing incidence X-ray diffraction (GIXRD) of 20 nm thick films (SnO_2_, MoS_2_) deposited on Si (100) wafers (Fig. 3) indicates that both materials are amorphous as grown by ALD at low temperatures (a-SnO_2_ and a-MoS_2_), which is in agreement with literature data for the ALD parameters applied here.^27,32^ Here, we will only deal with amorphous MoS_2_, since its electrocatalytic properties are superior to those of crystalline MoS_2_. Tin(iv) oxide, however, crystallizes to cassiterite upon annealing at 400 °C for 4 h (c-SnO_2_, COD 1000062). XRD performed in regular Bragg–Brentano geometry on the functional electrodes confirms the formation of c-SnO_2_, with additional diffraction patterns for Ni and Al (COD 2102278, COD 2300250, Fig. S3†).

**Fig. 4:**
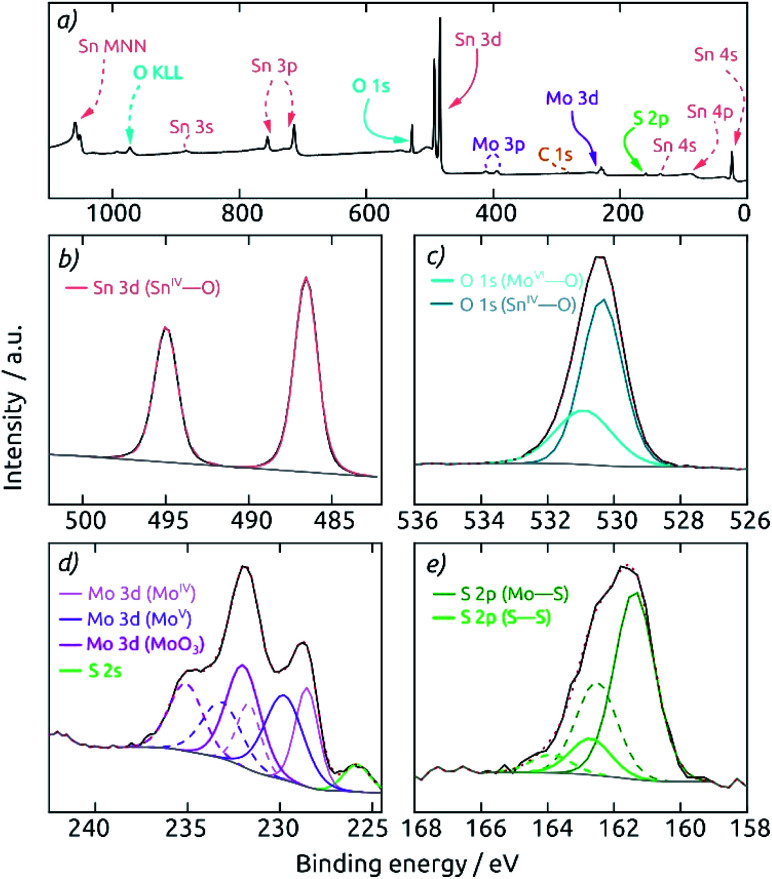
X-ray photoelectron spectra of a macroporous AAO/c-SnO_2_/a-MoS_2_ composite electrode after 30 s sputter-etching. The spectra were calibrated to a C 1s binding energy of 284.8 eV. (a) Survey spectrum showing the expected elements O, Sn, Mo, and S. Deconvolution of the core level spectra of the individual elements: (b) Sn 3d (here, a linear background was used to fit the data), (c) O 1s, (d) Mo 3d, and (e) S 2p. The deconvolution is shown with individual peaks in the element's characteristic color (doublet peaks in dashed lines). The measured spectrum is displayed in black, the background in gray and the envelope in red (dotted).

A highly surface-sensitive information is provided by XPS. The survey XPS spectrum of a AAO/c-SnO_2_/a-MoS_2_ composite electrode features (after a short sputter treatment) only the expected signals of O, Sn, Mo and S (Fig. 4a). No signal for Al is observed, indicating the absence of any pinholes across the c-SnO_2_/a-MoS_2_ coating. The Sn 3d region (Fig. 4b) displays two peaks at 486.6 eV and 495.0 eV, corresponding to 3d_5/2_ and 3d_3/2_ signals of Sn^IV^, respectively. The O 1s spectrum reveals two different species at binding energies of 530.4 and 530.9 eV, which can be attributed to O bound to Sn and Mo, respectively (Fig. 4c). The deconvolution of the Mo 3d region is displayed in Fig. 4d. Here, three different Mo species contribute to the spectrum. MoS_2_ is found with Mo 3d_5/2_ and Mo 3d_3/2_ peaks at 228.5 and 231.7 eV. A Mo^V^ species, which is mainly found in edge states of nanocrystalline molybdenum sulfides,^9,39^ is found at binding energies 229.8 and 233.1 eV for Mo 3d_5/2_ and Mo 3d_3/2_, respectively. The doublet at 232.0 and 235.1 eV is characteristic of MoO_3_ generated upon superficial oxidation of MoS_2_ in contact with air. The S 2s peak overlaps with the Mo signals at 225.8 eV. The S 2p region, which is well separated from the rest, can be deconvoluted into two different S doublets (Fig. 4e). The main component is S present in MoS_2_ with peaks at 161.4 and 162.5 eV for S 2p_3/2_ and S 2p_1/2_, respectively. Peaks at higher binding energies of 162.7 and 163.9 eV correspond to the S 2p_3/2_ and S 2p_1/2_ contributions of the desired, catalytically active S–S bound edge species.^9,40^

### Electrochemical investigation: performance and stability

Cyclic voltammograms are used in the range of +0.2 V to −0.7 V (*vs.* SHE) to compare the cathodic hydrogen evolution performance of electrodes featuring various geometric parameters. The comparison of various pore lengths allows one to observe corresponding capacitive contributions, proving that the full surface of the pores is in contact with the electrolyte (Fig. S4a†). However, the pore length does not improve the HER performance in any significant manner, indicating a transport limitation for the reaction (Fig. S4b†).

**Fig. 5:**
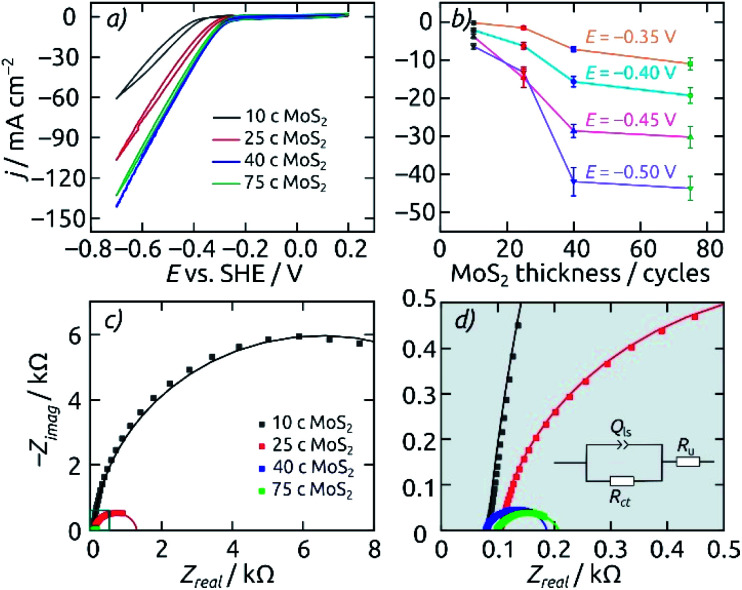
Influence of different MoS_2_ loadings on the electrocatalytic HER performance of AAO/c-SnO_2_/a-MoS_2_ composite electrodes. All electrodes feature pore lengths of 8.6 μm and a 10 nm thick annealed layer of SnO_2_. (a) Cyclic voltammograms for catalyst loadings of 10 c (black), 25 c (red), 40 c (blue) and 75 c (green), from +0.2 V to −0.7 V (*vs.* SHE). Scan rate 50 mV s^−1^, step size 2 mV. (b) Average steady-state current densities at various applied potentials. (c) Nyquist plots for the same electrodes recorded at −0.3 V (*vs.* SHE). Fitted curves are displayed as solid lines. (d) Close-up of the EIS data near the origin and equivalent circuit model used for the fit. All measurements are performed with an electrode featuring a macroscopic area of 0.0314 cm^2^ in 0.1 M H_2_SO_4_ as the electrolyte.

Fixing the pore length at 8.6 μm, the effect of electrocatalyst loading in the AAO/c-SnO_2_/a-MoS_2_ system can be highlighted (Fig. 5a). Upon increasing the MoS_2_ catalyst amount from 10 ALD cycles (10 c) to 25 c and 40 c, the HER onset potential (defined as potential required to achieve a cathodic current density of −10 mA cm^−2^) is reduced from −0.45 V to −0.34 V and −0.31 V (*vs.* SHE), respectively. However, a catalyst layer thickness further increased to 75 c of MoS_2_ delivers no significant additional benefit. If the steady-state current densities (averaged over 30 minutes, after a 60 minute electrolysis at each potential) are considered at various set potentials instead, a similar picture is obtained: 40 c of MoS_2_ ALD represents the most efficient use of the catalyst. Electrochemical impedance spectroscopy performed at −0.3 V (*vs.* SHE) corroborates these results. The Nyquist plots and their fits are presented in Fig. 5c/d. The charge transfer resistance *R*_ct_, which is associated with the catalytic activity, decreases from 13 to 1.2 and 0.11 kΩ when the MoS_2_ layer thickness increases from 10 to 25 and 40 c. Performance then plateaus out, with the thicker MoS_2_ layer (75 c) still at 0.11 kΩ (3.5 Ω cm^2^ for a circular sample of 2 mm diameter). Thus, a catalyst loading of 40 c MoS_2_ is set as the standard for subsequent electrode characterization.

**Fig. 6:**
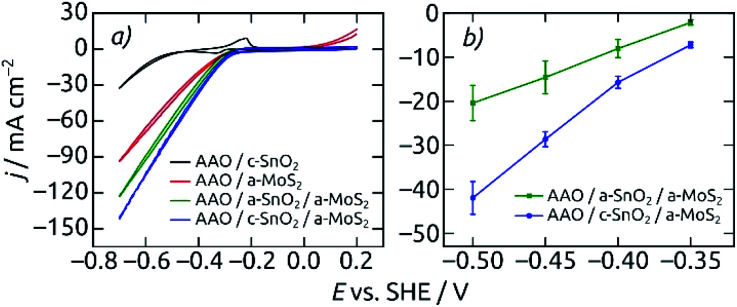
Electrocatalytic performance for different electrode compositions. Macroporous AAO coated with only c-SnO_2_ (black) and only MoS_2_ (red) are compared to AAO/SnO_2_/a-MoS_2_ composite electrodes featuring an amorphous (a-SnO_2_, green) or crystalline (c-SnO_2_, blue) tin oxide underlayer. (a) Cyclic voltammograms, measured from +0.2 V to −0.7 V (*vs.* SHE). Scan rate 50 mV s^−1^, step size 2 mV. (b) Average steady-state current densities *vs.* applied potential for AAO/SnO_2_/a-MoS_2_ composite electrodes. The electrodes feature pore lengths of 8.6 μm and film thicknesses of 10 nm SnO_2_ and 40 c of MoS_2_. Macroscopic electrode area 0.0314 cm^2^, performed in 0.1 M H_2_SO_4_ electrolyte.

The annealed underlying SnO_2_ layer proves to be beneficial the electrocatalytic activity (Fig. 6). Both the CV curves and steady-state current density values at different applied potentials exhibit the best electrochemical performance for the AAO/c-SnO_2_/a-MoS_2_ configuration as compared to all other reference samples, with an HER onset potential of −0.31 V (*vs.* SHE). The improved performance with c-SnO_2_ as compared with a-SnO_2_ can be due to either the increased surface area generated by a rougher crystalline underlayer or the improved electrical transport characteristics offered by c-SnO_2_ (or a combination thereof). The limiting property of electrical transport along the elongated pore structure is substantiated by the linear (as opposed to exponential) shape of the voltammetry curves. It is further proven by the poor activity of the MoS_2_-coated samples bereft of SnO_2_.

Nanostructured electrodes having c-SnO_2_ in direct contact with the electrolyte feature a very characteristic voltammetric pair of waves at −0.33 V and −0.23 V associated with the corrosion of SnO_2_. The absence of these peaks in CVs for electrodes with an AAO/SnO_2_/a-MoS_2_ composition indicates that MoS_2_ perfectly covers the entire electrode with a continuous, pinhole-free layer, and thereby prevents SnO_2_ corrosion in the electrolyte, at least initially.

**Fig. 7:**
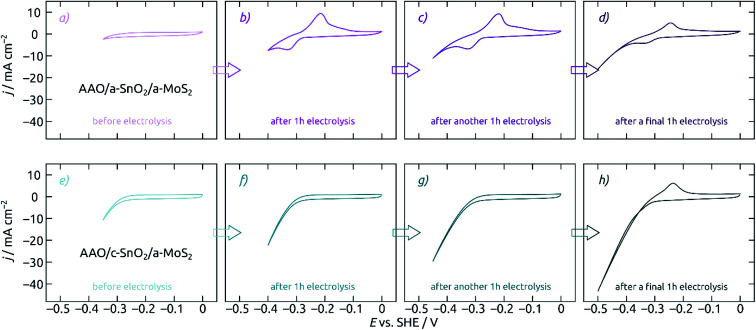
Influence of SnO_2_ crystallinity on the stability of AAO/SnO_2_/a-MoS_2_ composite electrodes. All electrodes feature pore lengths of 8.6 μm and 10 nm of SnO_2_ and MoS_2_. Cyclic voltammograms for electrodes with an as-deposited a-SnO_2_ coating (purple, a–d) and with annealed c-SnO_2_ (cyan, e–h) are characterized at different points of the electrochemical measurement sequence: (a, e) before electrolysis, (b, f) after 1 h at an −0.35 V (*vs.* SHE), (c, g) after another 1 h at −0.40 V, (d, h) after a final 1 h at −0.45 V. Scan rate 50 mV s^−1^, step size 2 mV, electrode area 0.0314 cm^2^. Performed in 0.1 M H_2_SO_4_ electrolyte. Not only does the annealed SnO_2_ underlayer give rise to a slightly improved performance, it also improves the stability very significantly.

Let us now characterize the stability of MoS_2_-based electrodes upon steady-state electrolysis. The experiment reported on in Fig. 7 consists in a series of electrolyses, each 60 minutes long, at increasingly negative potentials. One CV is presented before electrolysis (Fig. 7a/e), after an hour at −0.35 V (Fig. 7b/f), after an additional hour at −0.40 V (Fig. 7c/g), and finally after the last hour at −0.45 V (Fig. 7d/h). The evolution of the CV curve shapes is very different for the MoS_2_ electrode on a-SnO_2_ (Fig. 7a–d) and on c-SnO_2_ (Fig. 7e–h). The poor performance of the former is matched by its poor stability. The characteristic signals of SnO_2_, which are absent from the pristine sample's data (Fig. 7a), appear immediately upon electrolysis (Fig. 7b). The c-SnO_2_/a-MoS_2_ sample, however, remains perfectly stable up to −0.40 V (Fig. 7g), and only the relatively harsh potential −0.45 V proves to be deleterious for it (Fig. 7h, in 0.1 M H_2_SO_4_).

**Fig. 8:**
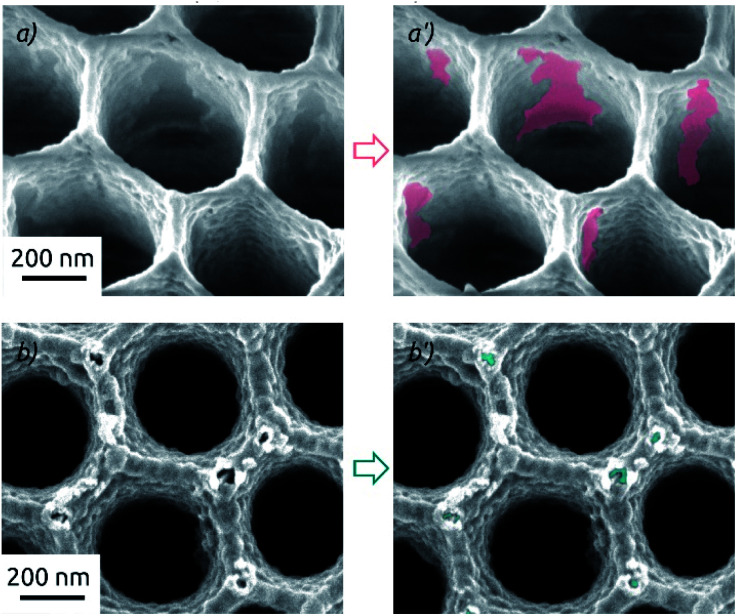
Scanning electron micrographs of macroporous electrodes after electrochemical HER (total 4 h at different applied overpotentials) featuring different layer compositions: (a and a′) AAO/a-SnO_2_/a-MoS_2_ (10 nm as-deposited SnO_2_). (b and b′) AAO/c-SnO_2_/a-MoS_2_ (10 nm annealed SnO_2_). All electrodes feature 40 c of MoS_2_, electrochemistry performed in 0.1 M H_2_SO_4_.

This drastic difference in the long-term behavior of samples prepared on a-SnO_2_ and c-SnO_2_ is highly reproducible. Its cause is apparent in scanning electron microscopic investigation of samples after the full 4 h of electrolysis stability investigation (Fig. 8). Both samples exhibit changes of the surface morphology after electrochemistry (see also Fig. 2a for comparison). Both samples experience a significant roughening, but the adhesion between MoS_2_ and the underlying SnO_2_ is profoundly different. On a-SnO_2_ (Fig. 8a), large areas of oxide have lost their MoS_2_ cover and are apparent. In other words, the MoS_2_ must have detached (areas highlighted in the micrograph). The MoS_2_ on the crystalline layer behaves much better: it maintains its continuity, apart from the appearance of individual, narrow corrosion pits at the protruding edges of the nanostructured surface (highlighted in Fig. 8b). These observations are perfectly in line with the contrasting stabilities observed in our electrochemical study. They indicate that the adhesion of MoS_2_ is significantly enhanced by the crystalline substrate.

## Conclusions

In conclusion, we establish a model electrode system in which not only the performance of MoS_2_ as an electrocatalyst but also its stability can be studied systematically as they depend on the chemical identity, crystallinity, and geometrical features of the porous substrate. The importance of an electrically conductive substrate is confirmed by the linear shape of cyclic voltammograms, which justifies the use of SnO_2_ as the substrate. The a-MoS_2_ deposited by ALD generates a perfectly continuous coating as soon as the layer thickness reaches a nominal value of 10 nm.

The performance of our samples can be quantified by the overpotential −0.25 V needed to reach 10 mA cm^−2^ in 0.1 M H_2_SO_4_, or −0.22 V in 0.5 M H_2_SO_4_ (values without any internal resistance compensation). The corresponding loading of amorphous MoS_2_ is 0.16 mg cm^−2^. Both of these values are comparable to the best featured in the literature (Table S2†).^9,11,40–46^ What the system presented here offers as a significant advantage is its control over the electrocatalyst stability. The agreement in the MoS_2_ electrocatalysis community is that a-MoS_2_ yields the best performance and c-MoS_2_ the best stability. Our proposal is that a-MoS_2_ on an appropriate crystalline surface such as c-SnO_2_ combines both advantageous properties—in addition to the electrical conductivity that is also required simultaneously.

Perhaps most importantly, we contend that this strategy might be of interest beyond the specific materials system studied here. Indeed, adhesion is an issue inherently associated with all 2D materials and represents a significant limitation as soon as bubbles of gaseous products generated by electrolysis impose mechanical constraints on the surface.^47,48^ This approach could further enhance the interest in transition metal dichalcogenide electrocatalysts, its generality remains however to be tested.

## Author contributions

JE and YC performed most of the investigation and wrote the original draft. SB provided SEM data and MKSB provided XPS data. EAQ and SC provided the Mo precursor and contributed to manuscript review. JB contributed conceptualization and manuscript review and editing. JE, YC and JB performed data curation.

## Conflicts of interest

There are no conflicts to declare.

## Supplementary Material

RA-011-D1RA00877C-s001
