# Supplementary material for: Stabilizing an ultrathin MoS2 layer during electrocatalytic hydrogen evolution with a crystalline SnO2 underlayer
Source: RSC Adv. 2021 May 18;11(29):17985–92. doi: 10.1039/d1ra00877c (PMC8129885; doi:10.1039/d1ra00877c)
Supplement: RA-011-D1RA00877C-s001 [file RA-011-D1RA00877C-s001.pdf]

Electronic Supplementary Material (ESI) for RSC Advances.
This journal is © The Royal Society of Chemistry 2021

Stabilizing an ultrathin MoS_2 layer during electrocatalytic hydrogen evolution with a crystalline SnO_2 underlayer

Supporting Information

Jonas Englhard,[‡,1] Yuanyuan Cao,[‡,1] Sebastian Bochmann,[1] Maïssa K.S. Barr,[1] Elsje Alessandra Quadrelli,[2] Julien Bachmann[1,3]*

1. *Chemistry of Thin Film Materials, Department of Chemistry and Pharmacy, IZNF, Friedrich-Alexander University of Erlangen-Nürnberg, Cauerstr. 3, 91058 Erlangen, Germany*
2. *C2P2 UMR 5265, Université de Lyon, Institut de Chimie de Lyon, CNRS, Université Lyon 1, ESCPE Lyon, 43 Bd. du 11 Novembre 1918, 69616 Villeurbanne, France*
3. *Institute of Chemistry, Saint Petersburg State University, Universitetskii pr. 26, 198504 St. Petersburg, Russia*

[‡] shared first authorship

** corresponding author: julien.bachmann@fau.de*

Keywords: atomic layer deposition, anodic alumina, tin oxide, molybdenum sulfide, hydrogen evolution reaction, water splitting

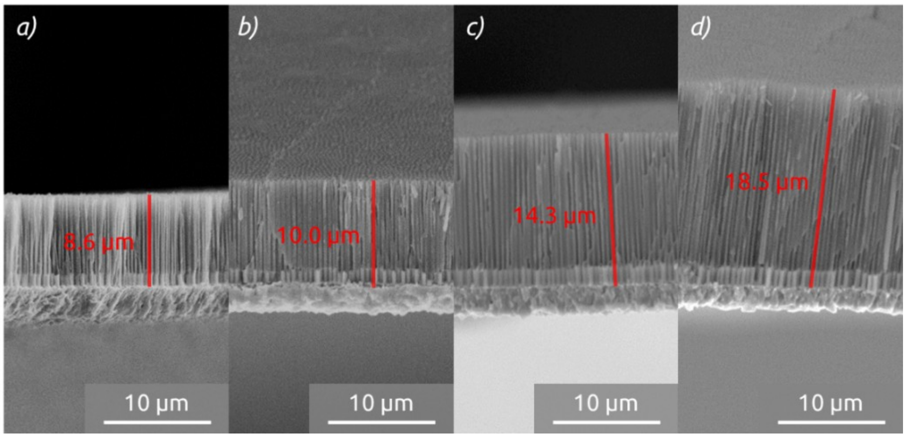

Figure S1. Cross-section scanning electron micrographs of AAO membranes anodized for *a)* 3 h, *b)* 4 h, *c)* 6 h or *d)* 8 h in 1 wt-% phosphoric acid at 195 V.

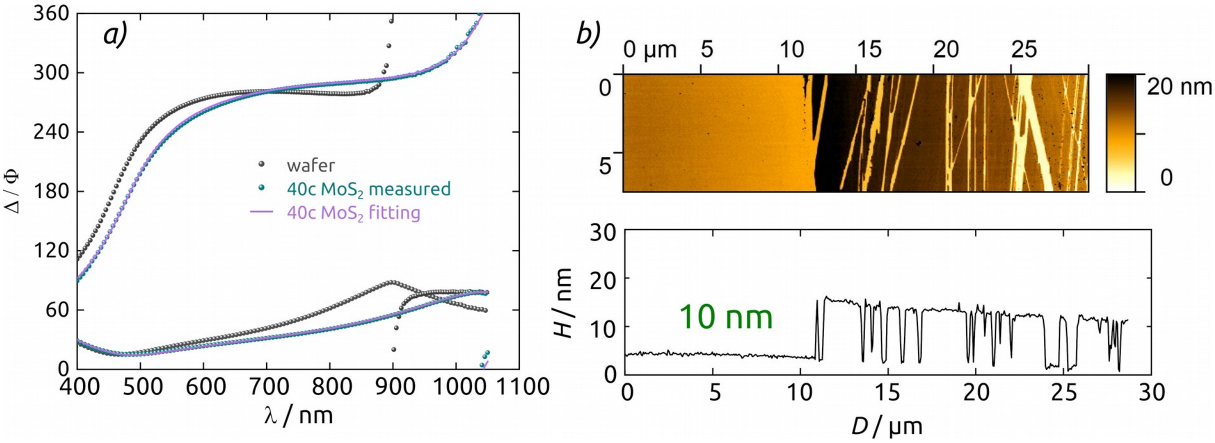

Figure S2. Thickness characterization of 40 c atomic layer deposited MoS_2 films. *a)* Spectroscopic ellipsometry: measured and fitted curves in dots and solid line, respectively; the fit yields an MoS_2 thickness of 9 nm. *b)* Step edge measurement by AFM, yielding a thickness of 10 nm.

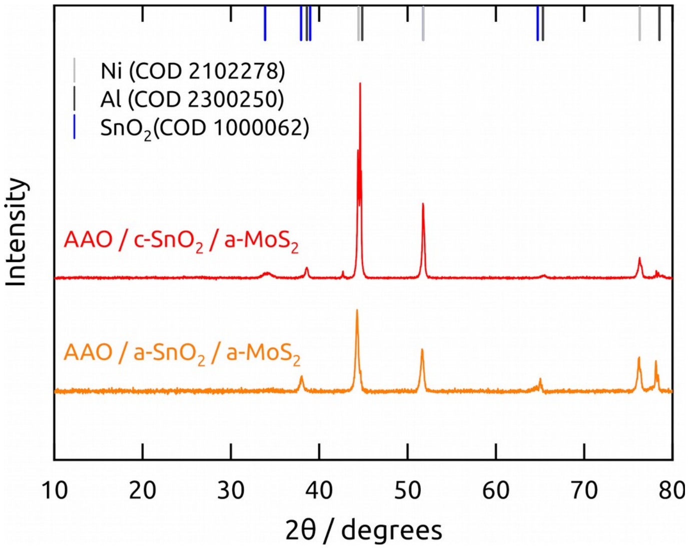

Figure S3. X-ray diffraction pattern of AAO / SnO_2 / MoS_2 composite electrodes. Comparison of samples with an as-deposited SnO_2 film (orange) and an annealed SnO_2^T film (red). All electrodes feature 10 nm SnO_2,40 c of MoS_2 and pore lengths of 18.5 µm. Diffraction peaks corresponding to Al (from frame; COD 2300250) and Ni (from backside-contact; COD 2102278) were observed for both samples. The annealed sample features diffraction peaks corresponding to crystalline cassiterite (COD 1000062). No crystalline MoS_2 was observed.

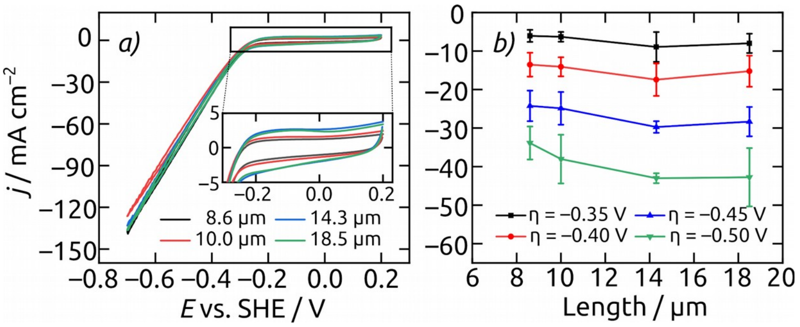

Figure S4. Electrocatalytic performance of AAO / c-SnO_2 / MoS_2 composite electrodes for different pore lengths: 8.6 µm (black), 10.0 µm (red), 14.3 µm (blue) and 18.5 µm (green). a) Cyclic voltammograms, measured from +0.2 V to –0.7 V (vs. SHE), scan rate 50 mV/s, step size 2 mV. *b)* Average steady-state current densities vs. pore length for various applied potentials (vs. SHE). The electrodes feature a 20 nm thick c-SnO_2 layer and 40 c of MoS_2. Electrode area 0.0314 cm^2, performed in 0.1 M H_2SO_4 electrolyte.

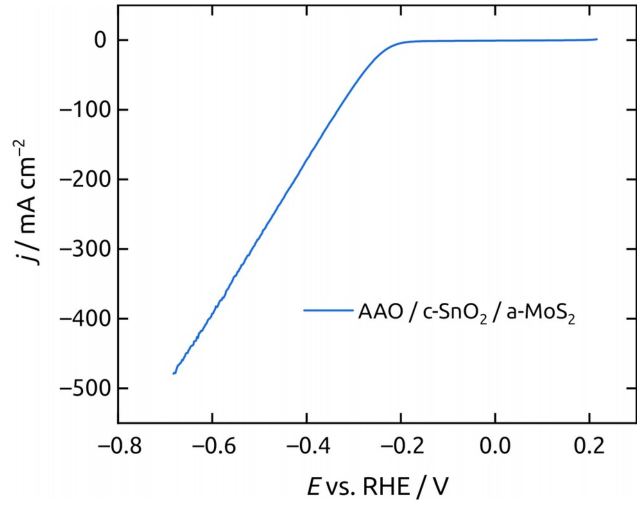

Figure S5. Linear sweep voltammogram for an AAO / c-SnO_2 / a-MoS_2 composite electrode featuring pore lengths of 8.6 µm, a 20 nm c-SnO_2 film and 40 c of MoS_2, recorded from +0.22 V to −0.68 V (vs. RHE). Scan rate 50 mV/s, step size 2 mV, electrode area 0.0314 cm^2. Performed in 0.5 M H_2SO_4 electrolyte.

Table S1. EIS fit parameters. All measurements performed in 0.1 M H_2SO_4 electrolyte.

**MoS_2 thickness / ALD cycles**	**R_u / Ω**	**R_{ct} / Ω**	**α_{ls}**	**Y_{ls} / $10^{-6}Ss^{\alpha}$**
10	82.5	12900	0.949	103
25	104	1210	0.921	303
40	79.8	107	0.896	824
75	98.7	109	0.789	1130

Table S2. Comparison of the HER activities of different amorphous MoS_2 based electrocatalysts.

Electrodes	Loading (mg/cm^2)	Current density (mA/cm^2)	Overpot. (mV)	Electrolyte (H_2SO_4)	preparation (catalysts)	Ref. in main text
MoS_x/PDOPA@ MWCNTs[a]	0.283	10	214	0.5 M	Wet chemical	[11]
MoS_3	0.2	10	160	1 M	Electrodeposition	[41]
MoS_6 AD[b]	0.1	10	161[c]	0.5 M	Electrodeposition	[42]
MoS_x QD[d]	----	5	800	0.5 M	Wet chemical	[40]
MoS_2/ graphene foam	----	10	264	0.5 M	Atomic layer deposition	[43]
MoS_x/N-doped CNT[e]	----	10	110	0.5 M	Wet chemical	[46]
MoS_2/TNTAs[f]	1.2	10	189	0.5 M	Atomic layer deposition	[9]
a-MoS_x/PbTe QD[d]/TNA[f]	4.5	10	138[c]	0.5 M	Electrochemical anodization	[44]
MoS_x	0.05	10	210	0.5 M	Wet chemical	[45]
MoS_2/SnO_2 /AAO[g]	0.16[h]	10	220	0.5 M	Atomic layer deposition	**This work**

[a] polydihydroxyphenylalanine (PDOPA), multiwalled carbon nanotubes (MWCNTs), [b] anodic deposition, [c] resistance-corrected, [d] quantum dots, [e] carbon nanotubes, [f] titanium oxide nanotubes arrays, [g] anodic aluminum oxide, [h] The catalyst loading was calculated assuming a perfect hexagonal structure with an edge length of 310 nm, a pore diameter after SnO_2 ALD of 340 nm and pore lengths of 7.5 μm (averaged values, determined by SEM).
